# Nonexercise Activity Thermogenesis is Significantly Lower in Type 2 Diabetic Patients With Mental Disorders Than in Those Without Mental Disorders: A Cross-sectional Study

**DOI:** 10.1097/MD.0000000000002517

**Published:** 2016-01-15

**Authors:** Hidetaka Hamasaki, Osamu Ezaki, Hidekatsu Yanai

**Affiliations:** From the Department of Internal Medicine, National Center for Global Health and Medicine Kohnodai Hospital, Chiba, Japan (HH and HY); and the Institute of Women's Health Science, Showa Women's University, Tokyo, Japan (OE).

## Abstract

Physical activity improves health in patients with mental disorders. Nonexercise activity thermogenesis (NEAT) represents energy expenditure due to daily physical activities other than volitional exercise. We aimed to evaluate NEAT in type 2 diabetic patients with and without accompanying mental disorders.

Between September 2010 and September 2014, we studied 150 patients with type 2 diabetes, 50 of whom also had a diagnosis of mental disorder, such as schizophrenia or mood disorder. We evaluated their NEAT in structured interviews using a validated questionnaire, and investigated differences in NEAT score and metabolic parameters between patients with and without mental disorders.

The NEAT score was significantly lower in patients with mental disorders than in those without (56.3 ± 9.9 vs 61.9 ± 12.1; *P* = 0.005). Patients with mental disorders had significantly higher triglyceride (184.5 ± 116.3 vs 146.4 ± 78.4 mg/dL; *P* = 0.02) and insulin levels (18.7 ± 20.1 vs 11.2 ± 8.5 μU/mL; *P* = 0.006), and significantly lower B-type natriuretic peptide (12.1 ± 13.3 vs 26.3 ± 24.8 pg/mL; *P* < 0.001) and brachial-ankle pulse wave velocity levels (1501 ± 371 vs 1699 ± 367 cm/s; *P* = 0.003) than patients without mental disorders. In patients with schizophrenia, specifically, NEAT showed a negative correlation with hemoglobin A1c levels (*β* = −0.493, *P* = 0.031), and a positive correlation with high-density lipoprotein cholesterol (*β* = 0.519, *P* = 0.023) and B-type natriuretic peptide levels (*β* = 0.583, *P* = 0.02).

Our results suggest that NEAT may be beneficial for the management of obesity, insulin sensitivity, and lipid profiles in patients with mental disorders. Incorporating NEAT into interventions for type 2 diabetes in patients with mental disorders, especially schizophrenia, shows promise and warrants further investigation.

## INTRODUCTION

Physical inactivity increases risks of cardiovascular disease (CVD), type 2 diabetes, cancer, and all-cause mortality.^1,2^ Compared with the general population, CVD-related mortality is higher in schizophrenia and depression is more prevalent in patients with CVD, although physical activity is able to ameliorate both.^3,4^ It has been suggested that physical inactivity has a negative effect on psychiatric symptoms in patients with mental disorders, such as depression and schizophrenia.^5,6^ The prevalence of depression decreases by approximately 6% in individuals who engage in light physical activities 3 h or more per week compared with sedentary individuals.^5^ Additionally, objectively measured physical activity is associated with improvement of depressive symptoms in adolescents.^7^ In patients with schizophrenia, physical inactivity during leisure time is associated with impairment of physical health-related quality of life evaluated by SF-36 scale.^6^ Strong evidence exists that physical activity improves health in these patients; however, they are often known to be sedentary.^8–10^ McCreadie^11^ showed that patients with schizophrenia spend only about 6 hours per week taking part in physical activity, and approximately 40% of these patients considered themselves to be physically inactive. When patients with schizophrenia engage in physical activity interventions, their symptoms have been shown to improve, as they do for patients with symptoms of depression and anxiety disorder.^12–14^ Moreover, habitual physical activity was shown to reduce the incidence of depressive symptoms in community-dwelling older people.^15^ Hence, the promotion of daily physical activity is important not only for the prevention of CVD but also in the management of common mental disorders.

Nonexercise activity thermogenesis (NEAT) represents energy expenditure due to physical activities other than active sports-like exercise and resistance training,^16^ and is the main determinant of variability in total daily energy expenditure.^17^ NEAT varies substantially between individuals by up to 2000 kcal per day.^18^ Since most obese individuals are sedentary, NEAT accounts for a high percentage of energy expenditure in daily lives. As such, it plays a crucial role in treating obesity.^19^ Most psychiatric medications induce weight gain,^20^ and obesity is associated with mental disorders including depression.^21^ Psychiatric patients with obesity can develop metabolic disorders. Thus, NEAT has an important role in the management of obesity in patients with mental disorders. We previously demonstrated that NEAT shows a favorable association with CVD risk factors in patients with type 2 diabetes.^22,23^ Promoting NEAT among psychiatric patients with type 2 diabetes could encourage them to develop good strategies for improving CVD risk factors and reduce the difficulties they face in maintaining recommended exercise therapy in daily life.

To our knowledge, NEAT has not been evaluated in psychiatric patients with type 2 diabetes before. Therefore, this study compared NEAT in type 2 diabetic patients with and without mental disorders.

## METHODS

### Patients

Between September 2010 and September 2014, we recruited 150 individuals aged >20 years who visited the Department of Diabetes and Endocrinology at our hospital for the first time with newly or previously diagnosed type 2 diabetes (based on the Japanese diagnostic criteria for type 2 diabetes). Mental disorders were diagnosed by skilled psychiatrists according to Diagnostic and Statistical Manual of Mental Disorder-4rth Edition or International Statistical Classification of Diseases and Related Health Problems 10th Revision criteria. Exclusion criteria were type 1 diabetes, severe physical disability such as cerebral infarction sequelae, and regular active sports-like exercise and resistance training. Information about medical history, smoking status, drinking habits, and medication was obtained by interview.

The study protocol was approved by the Medical Ethics Committee of National Center for Global Health and Medicine (reference number NCGM-G-001562-01), and the study was performed in accordance with the Declaration of Helsinki. All participants gave written informed consent for their data to be used in the study.

### Anthropometric and Physiological Measurements

Height and weight measurements were made using a rigid stadiometer and scales (DP-7100PW, Yamato Co., Ltd, Hyogo, Japan). Body mass index (BMI) was calculated as the body weight in kilograms divided by the height in meters squared. Waist circumference around the navel was measured with a nylon anthropometric tape with patients standing and exhaling. Blood pressure was measured using an automatic sphygmomanometer (HEM-762, Omron Co., Ltd, Kyoto, Japan), with participants in a sitting position. Brachial-ankle pulse wave velocity (baPWV) was measured using a pulse pressure analyzer (BP-203RPE, Nihon Colin, Tokyo, Japan) as an index of arterial stiffness.

### NEAT Assessment

At the first medical examination, patients were asked about their physical activity habits in a structured interview. To assess NEAT, we used our previously reported validated NEAT questionnaire.^22,24^ Each questionnaire item was scored for level of habitual physical activity (1–3 points) and the scores were totaled to give the NEAT score. To examine the validity of the NEAT questionnaire in psychiatric patients, we measured daily physical activity level (PAL) of 19 participants with mental disorders by the triaxial accelerometer (Active Style Pro HJA-350IT, Omron Co., Ltd, Kyoto, Japan). Detailed information on evaluating PAL by the triaxial accelerometer were previously published.^24^ Briefly, the participants wore the accelerometer under free living for 7 consecutive days, except under bathing and sleeping. Basal metabolic rate was estimated from a multiple regression equation which is suited to Japanese individuals.^25^ Total energy expenditure was calculated by a manufactured regression equation using metabolic equivalent values assessed by the triaxial accelerometer. PAL was calculated by the following equation: PAL = total energy expenditure/basal metabolic rate.^24^

### Blood Examination

Blood samples were taken from the antecubital vein. We measured levels of plasma glucose (PG), triglycerides (TG), high-density lipoprotein-cholesterol (HDL-C), low-density lipoprotein-cholesterol (LDL-C), serum insulin, and hemoglobin A1c (HbA1c). Enzymatic methods were used to assess PG (Glucose Assay Kit; Wako Pure Chemical Industries, Osaka, Japan), TG (TG-LH; Wako Pure Chemical Industries), and HDL-C and LDL-C (Cholestest N HDL and Choletest LDL; Daiichi Pure Chemicals, Tokyo, Japan). Serum insulin and HbA1c were measured by automated enzyme-linked immunosorbent assays (E-test TOSOH II; Tosoh, Tokyo, Japan) and high-performance liquid chromatography (HA-8180; Arkray, Tokyo, Japan), respectively. Plasma B-type natriuretic peptide (BNP) levels were measured using a specific immunoradiometric assay for human BNP (ARCHITECT BNP-JP, Abbott Japan Co, Ltd, Tokyo, Japan).

### Statistical Analyses

Data are expressed as mean ± standard deviation (SD). The association between NEAT score, and clinical, biochemical, and physiological data was examined by Pearson correlation coefficient. Multiple regression analysis was also performed to test the independent correlations between NEAT score, and physical and biochemical data. Models were adjusted for the potential confounders of age, sex, BMI, smoking, and alcohol habits. Differences in NEAT score, and anthropometric, physiological, and biochemical parameters between patients with and without mental disorders were analyzed by Student *t* test. Differences in sex ratio, smoking, and drinking habits, and treatments between patients with and without mental disorders were determined using the chi-square test. Statistical analyses were performed using SPSS version 22 (IBM Co., Ltd, Chicago, IL). *P* values less than 0.05 were considered statistically significant.

## RESULTS

### Patients

Table 1 shows the demographic characteristics of the patients (76 men and 74 women; age range 25–89 y). Fifty patients received a diagnosis of mental disorder: schizophrenia (50%), mood disorder (28%), and other (22%; alcoholic psychosis, neurosis, anxiety disorder, and sleep disorder). Patients with mental disorders were younger, more often women and smokers, and had diabetes of longer duration than those without mental disorders.

**TABLE 1:**
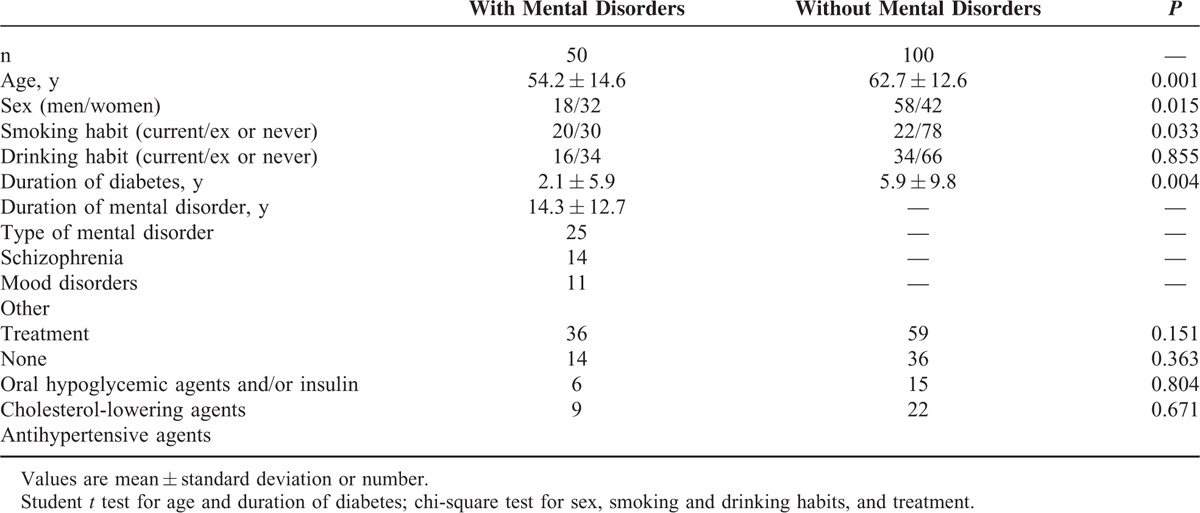
Characteristics of Patients With and Without Mental Disorders

### The Validity of the NEAT Questionnaire in Psychiatric Patients

**FIGURE 1:**
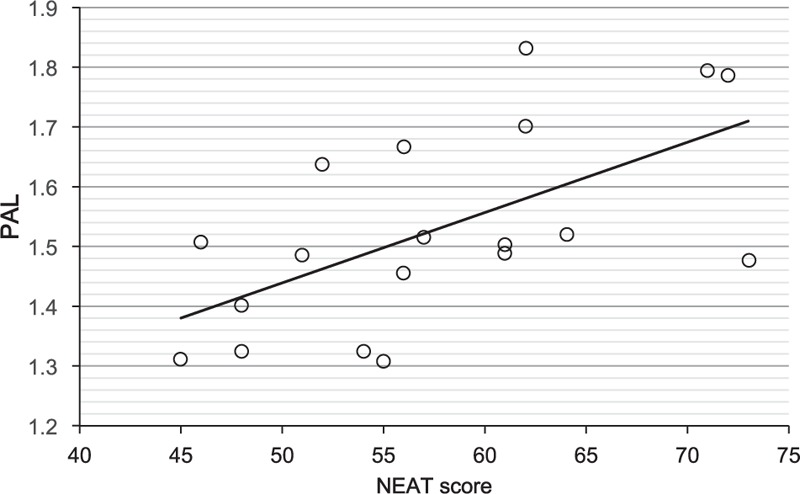
Correlation of nonexercise activity thermogenesis (NEAT) score and physical activity level (PAL) measured by the triaxial accelerometer.

The NEAT score in psychiatric patients was significantly and positively correlated with PAL measured by the triaxial accelerometer (*r* = 0.603, *P* = 0.006; Figure 1). This result was almost the same as the result of our previous study examining the validity of the NEAT questionnaire by comparing with objectively measured PAL by using the same triaxial accelerometer in 51 patients with type 2 diabetes.^24^

### Comparisons of NEAT Score and Physiological and Biochemical Parameters Between Patients With and Without Mental Disorders

The NEAT score was significantly lower by 9% in patients with mental disorders than in patients without. Weight, BMI, and waist circumference were significantly higher (by 18.7%, 18%, and 13.5%, respectively) in patients with mental disorders, indicating that psychiatric patients were more likely to be obese. No significant differences were observed in plasma glucose or HbA1c between patients with and without mental disorders. In patients with mental disorders, serum TG and insulin were significantly higher (by 26% and 67%, respectively), and plasma BNP and baPWV were significantly lower (by 54% and 11.7%, respectively) (Table 2).

**TABLE 2:**
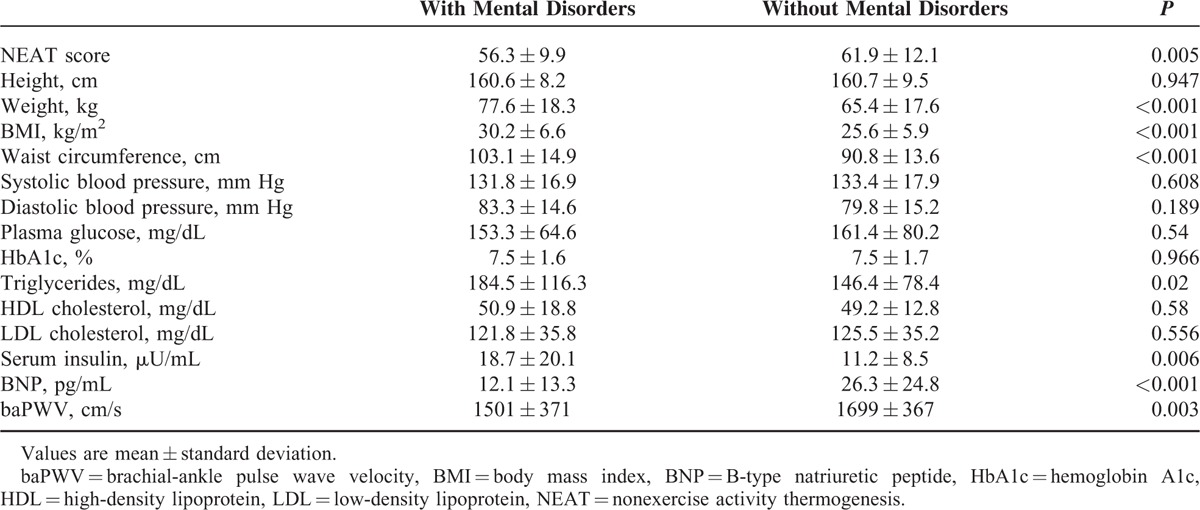
Comparison in the NEAT Score, Physiological and Biochemical Parameters Between Patients With and Without Metal Disorders

### Correlations Between NEAT Score and Metabolic Parameters

**FIGURE 2:**
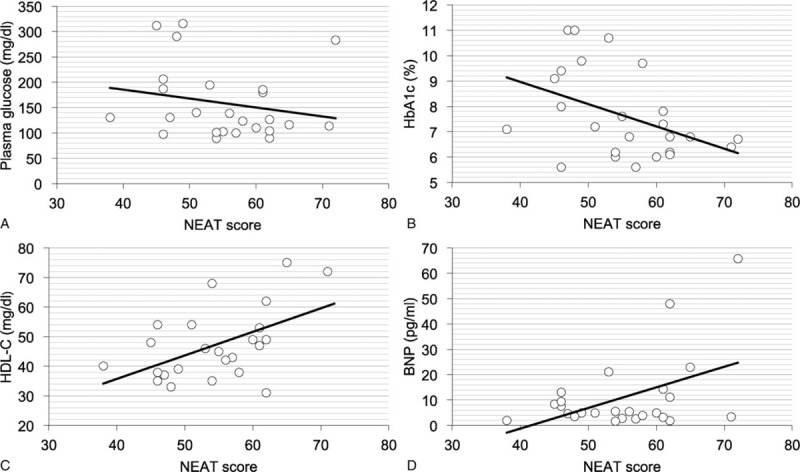
Correlation of nonexercise activity thermogenesis (NEAT) and levels of (A) plasma glucose, (B) hemoglobin A1c (HbA1c), (C) high-density lipoprotein cholesterol (HDL-C), and (D) B-type natriuretic peptide (BNP) in type 2 diabetic patients with schizophrenia.

In all patients, NEAT score showed an inverse correlation with waist circumference (*r* = −0.18, *P* = 0.044), serum TG (*r* = −0.17, *P* = 0.039), serum insulin (*r* = −0.263, *P* = 0.005), and duration of diabetes (*r* = −0.167, *P* = 0.042). After adjustment for age, sex, BMI, smoking and drinking habits, negative associations of NEAT score with waist circumference and duration of diabetes were not observed. However, NEAT score was significantly and inversely associated with serum TG (*β* = −0.178, *P* = 0.03) and serum insulin (*β* = −0.242, *P* = 0.003). In patients with mental disorder, NEAT score showed an inverse correlation with duration of diabetes (*r* = −0.339, *P* = 0.016), and there was a tendency toward a significant correlation between NEAT score and serum insulin (*r* = −0.32, *P* = 0.05). After adjustment for age, sex, BMI, and smoking and drinking habits, NEAT score was significantly and inversely associated with duration of diabetes (*β* = −0.329, *P* = 0.043); however, a significant association between NEAT score and serum insulin was not found (*β* = −0.359, *P* = 0.06). In patients with schizophrenia specifically, NEAT score showed a negative correlation with HbA1c (*r* = −0.429, *P* = 0.033), and a positive correlation with HDL-C (*r* = 0.514, *P* = 0.01) and plasma BNP (*r* = 0.454, *P* = 0.023) (Figure 2). After adjustment for age, sex, and BMI, NEAT score still showed a negative association with HbA1c (*β* = −0.493, *P* = 0.031), and a positive association with HDL-C (*β* = 0.519, *P* = 0.023) and plasma BNP (*β* = 0.583, *P* = 0.02).

## DISCUSSION

Recent meta-analyses have shown that physical activity improves the symptoms of depression, anxiety disorders, and schizophrenia in individuals with mental illness,^12–14^ but patients with schizophrenia and depression are often sedentary.^8–10^ An increase in physical activity in patients with mental disorders could reduce not only psychiatric symptoms, but also risk of CVD.

To our knowledge, this study demonstrates for the first time that psychiatric patients with type 2 diabetes have lower NEAT scores than nonpsychiatric patients with type 2 diabetes. In addition, our results suggest that NEAT may be beneficial for the management of obesity, insulin sensitivity, and lipid profiles in patients with mental disorders. NEAT score, which showed a negative correlation with duration of diabetes, was significantly lower in type 2 diabetic patients with mental disorders than in those without mental disorders, but the duration of diabetes was longer. Further, NEAT score was significantly lower in patients with mental disorders, in which the proportion of women was higher, than the patients without mental disorders, but the NEAT score was higher in women than in men among the study population as a whole (62.7 vs 57.4; *P* = 0.005, Student *t* test). Mental disorders have a strong influence on NEAT regardless of the duration of diabetes and sex difference. NEAT in patients with mental disorders is low because psychiatric patients tend to be more socially isolated and less motivated to be active in daily life and may be inactive due to their psychiatric symptoms. Such sedentary patients often tend to be obese and cannot engage in regular exercise. On the contrary, NEAT is not volitional exercise, has no considerable physical burden, and continues without cessation in daily life. Our results suggest that increasing NEAT will be important in the management of metabolic diseases in patient with mental disorders.

De Moor et al^26^ showed that low physical activity was associated with anxious and depressive symptoms in 5952 twins. They stated that the specific genes related to voluntary exercise behavior and risks for depression and anxiety are unknown, but the genes involved in the dopaminergic, norepinephrenergic, opioidergic, or serotonergic pathways of the brain are candidate genes that affect the regulation of physical activity and mood.^26^ In fact, dopamine is a crucial mediator in the regulation of spontaneous physical activity and NEAT.^27^ Low NEAT in patients with mental disorders could possibly be due to the action of these genes and their neuroendocrinological functions.

Higher levels of TG and insulin in patients with mental disorders could be caused by low NEAT in psychiatric patients. Daily physical activity improves insulin sensitivity and reduces TG levels,^28,29^ and NEAT may be associated with these favorable changes. Insulin resistance due to obesity should also be considered: obesity increases levels of intramyocellular TG in skeletal muscle, which impairs insulin sensitivity.^30^ In the present study, obesity-related parameters such as weight, BMI, and waist circumference were higher in patients with mental disorders than in those without, and these conditions might have increased the serum levels of TG and insulin. However, a causal relationship cannot be deduced; physical inactivity might have led to obesity in our patients with mental disorders, and low NEAT may be responsible for metabolic abnormalities in psychiatric patients.

Lower levels of BNP in patients with mental disorders might be explained by insulin resistance due to low NEAT. We previously reported that daily physical activity (NEAT) may increase plasma BNP levels and improve insulin resistance.^31^ Low NEAT may reduce insulin sensitivity, which in turn may decrease plasma BNP in patients with mental disorders. BNP levels increase with age and are inversely associated with BMI^32^; therefore, the lower levels in the patients with mental disorders may be due to younger age and higher BMI compared with those without mental disorders. However, its levels are also higher in women than in men,^33^ and the difference in BNP levels between type 2 diabetic patients with and without mental disorders might not have been affected by such demographic characteristics in this study. NEAT score showed a positive correlation with plasma BNP in patients with schizophrenia in this study. Natriuretic peptides mediate regulation of the hypothalamo-pituitary-adrenocortical system and their elevation may ameliorate anxiety symptoms.^34^ Ströhle et al^35^ reported that regular physical activity has anxiolytic effects mediated by atrial natriuretic peptide. There may be a relationship between NEAT and mental disorders mediated by BNP. However, the relationships between NEAT, insulin resistance, and BNP remain largely unknown. Further studies are warranted to reveal the association in psychiatric patients with type 2 diabetes.

Another novel finding of this study is that NEAT in patients with schizophrenia is favorably associated with plasma HbA1c levels. We previously reported favorable associations between NEAT and metabolic parameters in patients with type 2 diabetes or glucose intolerance^22,36^; however, no significant association was found between NEAT and plasma HbA1c; NEAT might not have been sufficient enough to elicit a beneficial effect on glycemic control in individuals without mental disorders. Therefore, in the present study, we investigated the differences in NEAT and other metabolic parameters between patients with schizophrenia or other mental disorders, and found that no significant differences exist. This result suggests that NEAT has a substantial role in glycemic control in patients with schizophrenia compared with individuals without schizophrenia. In patients with schizophrenia, whose physical activity levels are usually low, NEAT may have potential for improving long-term glycemic control.

The significant difference found in baPWV between patients with and without mental disorders seems to disagree with the favorable associations of NEAT with metabolic parameters. The number of smokers and serum TG levels were higher and NEAT score was lower; however, baPWV was lower in patients with mental disorders than in those without. Tomiyama et al^37^ reported that baPWV increases with age and is lower in women than men until age 60. In the present study, patients with mental disorders were significantly younger, more often women, and had shorter duration of diabetes than in those without mental disorders, and might have been the cause of this association between NEAT and baPWV. Although the effect of NEAT on arterial stiffness such as baPWV has not been reported to date, a systematic review and meta-analysis of randomized controlled trials showed that arterial stiffness was improved by high-intensity aerobic exercise.^38^ NEAT largely consists of low-intensity physical activities that may not be adequate enough to reduce arterial stiffness.

## STUDY LIMITATIONS

There are several limitations to this study. First, it was a cross-sectional study and this limits inferences of causality and its direction. While we cannot refer to the effects of NEAT on symptoms of mental disorders or on CVD risks, we did observe low NEAT in psychiatric patients with type 2 diabetes, which is consistent with the results of previous studies investigating physical activity in patients with mental disorders. Second, the sample size was relatively small and our results may not be applicable to other populations. Third, our NEAT questionnaire is a useful and reliable method of measuring NEAT,^24^ but it is possible that we are not evaluating the true level of NEAT and the results could be influenced by potential biases. To avoid biases regarding questionnaire method such as selection bias, the interview was performed by technicians at the Outpatient Clinic consecutively. However, other biases and confounding factors may exist. For example, overfeeding decreases NEAT,^39^ and regular high-intensity exercise increases NEAT,^40^ which suggest that dietary intake and sports-like activities influence the amount of NEAT. Although we controlled for confounding factors such as regular active sports-like exercise and drinking habits, total dietary intake was not taken into account. Genetic variations in study participants may also affect the results. Further studies are warranted. Fourth, the heterogeneities of the study population in terms of types of mental disorders and medication might have been confounding factors. However, schizophrenia and mood disorders accounted for about 80% of the psychiatric patients in this study, and no significant differences in treatment for type 2 diabetes, dyslipidemia, and hypertension were found, so the influence is likely to be small.

## CONCLUSIONS

We found that NEAT was significantly lower in patients with mental disorders than in those without. NEAT was favorably associated with TG and serum insulin levels, and also waist circumference. Furthermore, NEAT was inversely correlated with HbA1c levels in patients with schizophrenia. These findings suggest that NEAT can play an important role in the management of type 2 diabetes in patients with mental disorders, especially those with schizophrenia.
